# A hidden challenge: 2 novel endoscopic techniques to resect gastric exophytic subepithelial lesions

**DOI:** 10.1016/j.vgie.2025.12.002

**Published:** 2025-12-26

**Authors:** Farimah Fayyaz, Romina Roshanshad, Mouen Khashab

**Affiliations:** 1 Gastroenterology and Hepatology Division, Johns Hopkins Medicine, Baltimore, Maryland, USA; 2 Division of Gastroenterology, Department of Medicine, Chulalongkorn University, Bangkok, Thailand

## Abstract

**Background and Aims:**

Subepithelial gastric tumors originating from the muscularis propria, such as GI stromal tumors (GISTs), are challenging to remove endoscopically because of their deep location. Full-thickness resection (FTR) requires advanced endoscopic techniques to achieve complete and safe tumor removal.

**Methods:**

Two novel natural orifice translumenal endoscopic surgery (NOTES) techniques were applied for endoscopic resection of GISTs. In the first, a bidirectional FTR was performed using both luminal and peritoneal sides for resection. In the second, dissection was done from the peritoneal side while preserving mucosal integrity.

**Results:**

A 2-cm GIST was resected en bloc using bidirectional FTR. A second incidental 1.5-cm lesion discovered intraoperatively upon peritoneal exploration was resected using a serosal approach with mucosal preservation, requiring no closure. Full-thickness closure of the first resection site was achieved endoscopically with suturing systems.

**Conclusions:**

These 2 NOTES-based techniques allowed complete resection of exophytic GISTs without the need for surgical access. Bidirectional FTR permitted controlled dissection from both peritoneal and luminal sides, whereas the serosal approach preserved the mucosa and eliminated the need for closure. Together, these methods expand the endoscopic options for managing deep gastric subepithelial tumors.

## Introduction

GI stromal tumors (GISTs) are the most common mesenchymal neoplasms of the GI tract, with approximately 60% to 70% occurring in the stomach.^1^ Unlike most subepithelial tumors that arise from the submucosal layer, GISTs originate from the muscularis propria, making endoscopic resection technically challenging.^2^ Surgical resection remains standard for high-risk or larger GISTs, but minimally invasive endoscopic techniques are emerging as viable alternatives. Current endoscopic full-thickness resection (FTR) techniques have limitations when applied to exophytic lesions because they may not allow sufficient visualization of the tumor.^1^ Natural orifice translumenal endoscopic surgery (NOTES) offers transoral access to the peritoneal cavity, potentially avoiding abdominal incisions and their morbidity.^3^

**Figure 1:**
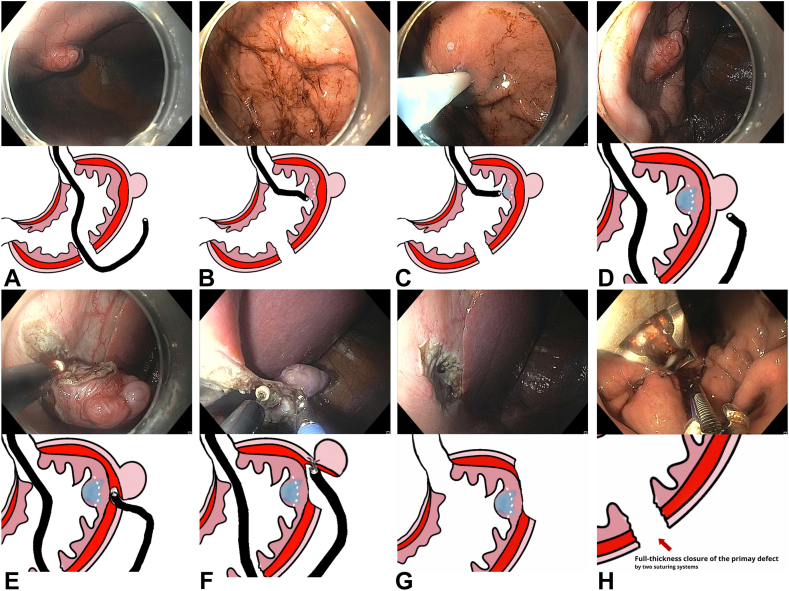
Serosal endoscopic dissection of the second exophytic lesion. **A,** A second lesion identified during peritoneal exploration. **B,** Lesion borders demarcation from the luminal side. **C,** Submucosal injection around the lesion from the luminal side. **D,** Blue dye around the lesion observed from the peritoneal side. **E,** Dissection of the lesion from the peritoneal side. **F,** Simultaneous grasp and resection of the lesion. **G,** Intact mucosa at the defect site. **H,** Full-thickness closure of the primary defect using suturing systems.

We describe a video case (Video 1, available online at www.videogie.org) in which 2 novel NOTES-based techniques were used to resect GISTs. The first technique is a variation of the bidirectional FTR previously described,^4^ involving dual access to the lesion via the gastric lumen and peritoneal entry. The second technique is a serosal-side dissection performed entirely within the peritoneal cavity, preserving the mucosa and achieving nonexposed resection (Fig. 1).

## Case presentation

**Figure 2:**
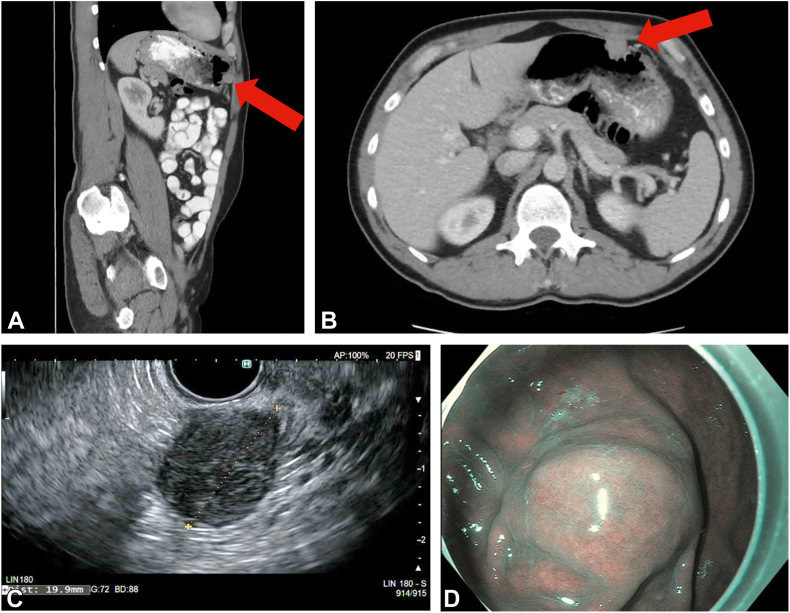
A 2-cm subepithelial lesion located in the anterior wall of the gastric body. **A,** Sagittal view of an abdominal CT scan showing the lesion (*arrow*). **B,** Axial view of the abdominal CT scan showing the lesion (*arrow*). **C,** EUS image of the lesion. **D,** Endoscopic view of the lesion.

**Figure 3:**
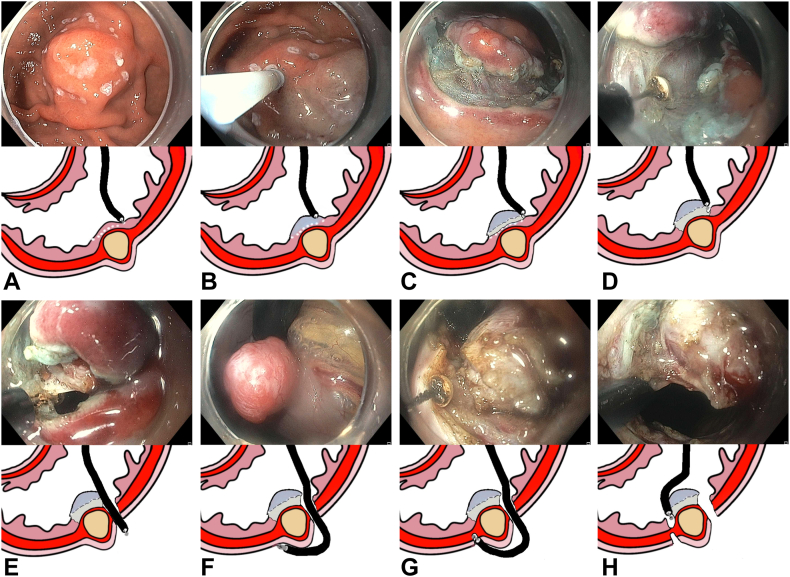
Bidirectional full-thickness resection of the primary GI stromal tumor. **A,** Demarcation of the lesion borders. **B,** Submucosal injection around the lesion. **C,** Circumferential incision around the lesion. **D,** Submucosal dissection. **E,** Full-thickness resection and entry into the peritoneal space. **F,** Coagulation of gastric wall vessels around the lesion from the peritoneal side. **G,** Full-thickness resection from the peritoneal side. **H,** Full-thickness resection from the luminal side.

A 46-year-old patient had a 2-cm subepithelial lesion in the anterior wall of the distal gastric body on abdominal CT (Fig. 2). EUS-guided fine-needle aspiration confirmed a GIST arising from the muscularis propria, and the patient was given the options of surgical management and endoscopic resection and opted for minimally invasive endoscopic resection. The surgical team was aware, and the procedure was performed in an inpatient hospital unit with surgical backup. The procedure was performed in the endoscopy suite with the patient in a supine position under general anesthesia with endotracheal intubation for airway protection, immobility, and controlled insufflation. Given the concern for potential contamination, intravenous antibiotics were administered at the start of the procedure. Carbon dioxide was used for insufflation for the duration of the procedure. A therapeutic gastroscope (GIF-HQ190; Olympus America, Center Valley, Pa, USA) was introduced. The lesion appeared as a well-defined subepithelial bulge. Borders were marked with a DualKnife J 2.0 mm (Olympus America) on soft coagulation. Submucosal injection was performed to achieve mucosal lift, followed by a circumferential incision. Submucosal dissection was performed using the DualKnife J and ITknife2 (Olympus America), exposing the tumor margins and muscle attachment. A full-thickness incision using the ITknife2 allowed the gastroscope to enter the peritoneal cavity. Using transillumination and under direct visualization, we identified a safe window for Veress needle entry through the anterior abdominal wall. The Veress needle was introduced and connected to a robotic insufflator (EVA15; Palliare, Inc, Oceanside, Calif, USA) to maintain peritoneal pressure at 12 to 14 mm Hg for the duration of the procedure. Gastric wall vessels around the lesion were coagulated from the peritoneal side. FTR was completed bidirectionally from both peritoneal and luminal sides, resulting in en bloc resection (Fig. 3).

Upon peritoneal exploration, which is a standard step of NOTES procedures to ensure no bleeding or injury to surrounding structures, a second 1.5-cm exophytic lesion was discovered on the serosal surface of the proximal gastric body along the greater curvature, not visualized on preoperative CT. Given the exophytic location, a serosal-side resection technique was chosen. From the gastric lumen, the corresponding region was marked circumferentially. Submucosal injection was then performed to move the lesion away from the mucosa, facilitating the dissection of the lesion off the muscle without mucosal injury. Dissection was done from the serosal aspect using the DualKnife J and ITknife2. The scope was exchanged for a dual-channel therapeutic scope (GIF-2TH180; Olympus America), allowing grasping with the Raptor Grasping Device-360 8.3 mm (STERIS, Mentor, Ohio, USA) and resection with the ITknife2. Once freed, the lesion was withdrawn into the stomach through the initial defect. Because the mucosa remained intact, no endoscopic closure was required for this second lesion. The primary defect was closed with 2 over-the-scope suturing systems (OverStitch; Boston Scientific, Marlborough, Mass, USA) (Fig. 1).

The patient was admitted overnight for observation and kept without oral intake. Prophylactic intravenous antibiotics were administered. A contrast esophagram obtained on postoperative day 1 showed no evidence of leak, and the patient was clinically doing well. After tolerating a liquid diet on the same day, he was discharged on a soft diet for 2 weeks. The patient was instructed to continue oral antibiotics for a total antibiotic therapy duration of 3 days.

The histopathology report of the primary tumor showed a 2.5-cm epithelioid-type GIST histology with mixed spindle cells, with negative resection margins. The second tumor was a 2.0-cm epithelioid-type GIST and appeared to be centered in the muscularis propria. The GIST appeared to be multifocal, exhibiting a multinodular/plexiform growth pattern. The mitotic rate was 1 per 5 mm^2^, and no tumor necrosis was identified.

Next-generation sequencing did not reveal typical mutations in KIT proto-oncogene receptor tyrosine kinase or platelet-derived growth factor receptor alpha. A variant of unknown significance was identified in succinate dehydrogenase complex subunit A, and germline genetic testing was planned. The patient has undergone CT surveillance every 3 months, and at 18 months after the procedure, no recurrence had been detected.

## Conclusions

Two novel NOTES techniques were used to resect GISTs. The first technique, bidirectional FTR, involves accessing the peritoneal cavity to resect the lesion from both the luminal and peritoneal sides, with potential advantages of reducing the risk of incomplete resection, providing an additional dimension of visualization, and minimizing the risk of injury to surrounding structures and blood vessels. The second technique involves dissection from the serosal aspect while preserving the mucosa, eliminating the need for endoscopic closure and enabling a no-exposure resection from the peritoneal side. These procedures are technically demanding and require familiarity with NOTES techniques, and tumor location is an important consideration, because accessibility from the peritoneal side varies across gastric regions. Together, these approaches demonstrate the feasibility of endoscopic management for muscularis propria tumors without surgical access.

## Patient Consent

Informed consent was obtained from the patient for the procedures performed, including discussion of risks and benefits, and for the use of images and recordings for research and publication purposes.

## Disclosure

The following author disclosed financial relationships: M. Khashab is a consultant for Boston Scientific, Olympus America, Medtronic, and MicroTech, and receives royalties from UpToDate and Elsevier. All other authors disclosed no financial relationships.

## Supplementary data

Video 1 Two novel endoscopic techniques to resect gastric exophytic subepithelial lesions.
